# Functional Vision Questionnaire Detects Near Triad Impairments in Adolescent Athletes With Concussion History

**DOI:** 10.1097/WNO.0000000000002275

**Published:** 2024-12-13

**Authors:** Jouko Leinonen, Roosa Mikkola, Kati Peltonen, Laura Hokkanen, Tiina Laitala

**Affiliations:** THAT (Tissue Healing In Trauma) group (JL, RM, TL), Institute of Biomedicine, University of Turku, Turku, Finland; and Department of Psychology and Logopedics (KP, LH), University of Helsinki, Helsinki, Finland.

## Abstract

**Background:**

Concussions are mild traumatic brain injuries that often cause vision problems. They have significant impacts on everyday life, cognitive capacity, and sports performance, and may affect injury prevalence in fast contact sports such as ice hockey. A functional vision questionnaire specifically designed for sports was used here to study the correlation between vision problems and concussion history.

**Methods:**

In this national cross-sectional concussion study, 860 Finnish elite-level male adolescent ice hockey players (aged 13–21 years) answered a functional vision questionnaire and performed a computerized neurocognitive test, ImPACT. Totally 265 athletes reported a history of at least 1 concussion. All data were statistically compared with age-matched athletes with no concussion history (n = 595). For further analysis, athletes were divided into subgroups by age and number of previous concussions.

**Results:**

Previously concussed athletes reported more general and eye-specific symptoms than their healthy controls. Increases in eye fatigue, frontal headaches, and blinking were statistically significant. Also statistically more problems with depth perception and evaluating distances, concentration problems, blurred vision, and losing the object in sight were observed among athletes with concussion history.

**Conclusions:**

Concussion history reflects an increase in the prevalence of vision deficits, as determined by multiple disturbances in the near triad. The significant number of vision problems in the concussion history groups strongly suggests that functional vision should routinely be evaluated in athletes. The vision problems observed in the athletes with concussion history may indicate an increased injury risk that should be addressed.

Concussion is a mild traumatic brain injury (mTBI), which results in the rapid onset of transient neurologic dysfunction. ICD-11^1^ defines concussion as “loss or diminution of consciousness due to injury.” There are no diagnostic imaging findings after concussion,^2^ because it causes rather metabolic than structural damages. Concussion is a common injury especially in contact sports such as ice hockey, with an incidence of 0.76/100 athlete exposures for men, and 0.76/1,000 athlete exposures for women.^3^ Although patients with concussion do not present significant morphologic brain pathology, diagnosis is symptom based.^4^ Imaging studies, however, suggest that already subconcussive rotational acceleration of the head leads to altered midbrain white matter integrity in healthy football players.^5^ Neuro-ophthalmic deficits are often observed acutely after concussion.^6^ Up to 95% of concussed children and adolescents present with at least 1 vision-related anomaly (accommodation problem, convergence insufficiency, and/or saccadic dysfunction).^7^

All brain lobes participate in vision, and several visual centers are located throughout the brain. These 3-dimensional neuronal networks run in anteroposterior, dorsoventral, and craniocaudal orientations.^8^ Visual stimuli are transported from the retina all the way to the occipital primary visual cortex, where image formation takes place. To initiate cognitive processing of the received visual data, interpretation begins at the cortical, interconnected ventral “what” and dorsal “where” visual pathways at the occipitotemporal and parietal cortexes.^9^ To maintain clear vision, horizontal and vertical gaze centers located in the brainstem control gaze shifting and holding together with motor nuclei of cranial nerves III, IV, and VI. Owing to the complex 3-dimensional neuroanatomy, visual tracts are sensitive to rotational, traction–compressional, and metabolic trauma caused by even subconcussive head impacts. Concussions do not damage the cranial nerves II, III, IV, or VI, but lead to a neurometabolic cascade: intracellular energy crisis, neuronal dysfunction, and neuroinflammatory response.^10,11^ Visual acuity remains normal, visual field defects and cranial nerve palsies are very rare, but headache, accommodation disorders, and convergence insufficiency are common.^12^

Although concussion symptoms usually resolve over the course of 2–4 weeks,^13^ up to one-third may have prolonged symptoms.^14^ Research has mainly concentrated on the acute prevalence of postconcussive vision problems, but studies on long-term effects of accumulating concussions on vision are missing. At least among children and adolescents, vision and vestibular problems are suggested to predict prolonged concussion recovery.^15^ Although the new version of Sport Concussion Assessment Tool, SCAT6,^16^ addresses double vision and extraocular eye movements, its aim is to find possible neurologic red flags that require hospitalization of the injured athlete, not to evaluate sport vision impairments.

Most of the studies on concussion-induced acute (0–2 weeks) and long-term (>4 weeks) changes in vision have been small population studies performed on mainly patients acutely concussed. This study approaches vision problems from a different angle by focusing on concussion history and current vision problems within a large adolescent male elite athlete population. To our knowledge, this is the first questionnaire-based cross-sectional study to correlate concussion history and vision dysfunctions in clinically healthy athletes. None of the athletes enrolled in this study were diagnosed with postconcussion syndrome or acute concussion, so our findings suggest that sport vision should be addressed in detail after each concussion to restore the complex neurologic circuits responsible for clear vision during active head movements.

## METHODS

### Participants

These data were collected as part of a 3-year concussion follow-up study “Heads in the Game” coordinated by the University of Helsinki, Finland.^17^ The project included extensive baseline testing and a structured program to identify concussions and monitor recovery in adolescent elite ice hockey players in Finland. The subjects for this study were voluntarily recruited from the participating teams, and answers to the functional vision questionnaire were received during off-season from a total of 926 athletes. In the study population, 265 athletes had a history of at least 1 concussion, while 595 were healthy controls. To minimize the limitation that self-reportion creates, the study was performed in the presence of health care professionals. This study was approved by the HUS Ethical Committee (Helsinki, Finland). Each participant and, if the athlete were younger than 16 years of age, a parent/guardian signed a voluntary informed consent. The study was conducted according to the Declaration of Helsinki.

### Procedures

The functional vision questionnaire used here was developed to identify and characterize possible vision problems that have an impact on sport performance. Some questions targeted general symptoms such as eye fatigue, headaches, and concentration problems, and others addressed functional sport vision (Table 1). Questions were designed so that the needs to compensate for possible vision dysfunctions, for example, blinking and squinting, could be observed. The participants answered the questions “*yes*” or “*no*” on their native language, and all data were digitalized for statistical testing. Data from the functional vision questionnaire were cross analyzed with ImPACT data to identify the athletes with a history of concussion. Of all the players who answered the questionnaire, 66 athletes did not attend the ImPACT baseline test, so these individuals were excluded from further analysis. This study, therefore, included 860 adolescent male athletes (aged 13–21 years). If an answer for a question in the vision questionnaire was missing or unclear, answer for that specific question was excluded.

**TABLE 1.** The functional vision questionnaire used in this study

1. Do you use eyeglasses?
2. Have you had problems seeing traffic signs or watching TV?
3. When watching far and near in turns, does it take a moment before the object is clear?
4. Do you have to squint to see far better?
5. Do you suspect that there is something unusual in your eyes or vision, even though you think you always see well?
6. Have your eyes been operated on?
7. Have you been told in an eye examination that you have strabismus?
8. Have you been given vision exercises at some point of your life?
9. Have you used eye glasses in childhood but stopped using them later?
10. Do you sometimes have difficulties with near vision?
11. After reading, is your vision blurry when you look into the distance?
12. Do your eyes get tired when you read or use a computer?
13. Does the text sometimes become blurry during reading?
14. Do you have to blink to make an unclear object become clear?
15. Do you take breaks during close-up work because of eyes strain?
16. Do you have problems concentrating when reading, for example, need to read again because you did not understand the text after the first reading?
17. Do you move the text closer or farther away when reading to see it better?
18. Do you have difficulties keeping your sight on the row, or moving to the next row, or do you feel that rows and letters jump and mix?
19. Do you sometimes close one eye while reading?
20. Have you experienced frontal headaches?
21. Are your eyes sensitive to light, or have they become light sensitive?
22. Do your eyes feel tired or strained at times?
23. Does it affect your sport performance, if you read or play on a computer before sport?
24. Do you have problems with your vision during sport?
25. Do you feel that your vision affects your sport performance?
26. Have you ever had difficulties in keeping your eyes on a moving object, for example, a ball or a puck?
27. Do you have difficulties in directing your sight on a moving object?
28. Do you at times lose the object in sight?
29. Do you have difficulties in observing the spinning of a ball or puck?
30. Have you ever had difficulties with depth perception, or in evaluating distances?
31. Have you ever had difficulties in knowing where the ball/puck or other players are?
32. Have you ever noticed blurring or disappearing of the borders of field of sight during sport performance?
33. Do you feel that you make the same mistakes repeatedly in your sport performance?

Athletes answered the questions “*yes*” or “*no*.” The answer “*yes*” was considered a pathologic reply.

### Statistical Analysis

All data analyses were performed using IBM SPSS Statistics software version 29.0 (IBM Corp, Armonk, NY). Differences between groups in the questionnaire were observed with χ^2^ tests and Fisher exact test. Normality of the data was checked by exploring skewness, kurtosis, histogram, and Q-Q-plot. The association between “*yes*” answers and number of previous concussions was evaluated by Pearson correlation test. Significance levels were set at *P* < 0.05.

## RESULTS

All athletes were healthy at the time of the study, and no acute concussions or athletes with postconcussion syndrome were enrolled in the study. Time from the last concussion varied between 45 days and 162 months (median 20 months). Statistical analysis confirmed that the time from the last concussion did not correlate with the answers given in the functional vision questionnaire. Therefore, any impairment in visual performance observed in the analysis was not due to acute concussion or recovery therefrom. A summary of the study population is shown in Table 2. Although our study population included 860 individuals of whom 265 athletes had experienced at least 1 concussion, the number of concussed athletes per age group remained low, specifically for the individuals with 3 or more concussions in their history. This was seen as nonstatistically significant trends in the data analysis.

**TABLE 2.** Concussion frequency and distribution of “*yes*” answers in the whole study population, and in subgroups divided by age

Athlete Group	Number of Concussions in the History	Number of Yes-Answers Given in the Functional Vision Questionnaire	n	%
All	0	2.81	595	69.2
	1	3.16	161	18.7
	2	3.44	68	7.9
	3	2.84	25	2.9
	≥4	4.27	11	1.3
		Total	860	100
13–14 y	0	3.01	78	75.0
	1	3.55	20	19.2
	2	3.6	5	4.8
	3	0	0	0
	≥4	1	1	0.96
		Total	104	100
15–16 y	0	2.81	273	70.2
	1	2.68	74	19.0
	2	3.26	27	6.9
	3	2.9	14	3.6
	≥4	2	1	0.26
		Total	389	100
17–18 y	0	2.78	171	69.8
	1	3.61	46	18.8
	2	3.05	21	8.6
	3	2.67	6	2.4
	≥4	7	1	0.41
		Total	245	100
19–21 y	0	2.63	73	59.8
	1	3.48	21	17.2
	2	4.27	15	12.3
	3	2.8	5	4.1
	≥4	4.63	8	6.6
		Total	122	100

There was a statistically significant correlation between the number of “*yes*” answers and number of concussions (*P* = 0.025; Pearson r = 0.077).

**FIG. 1.**
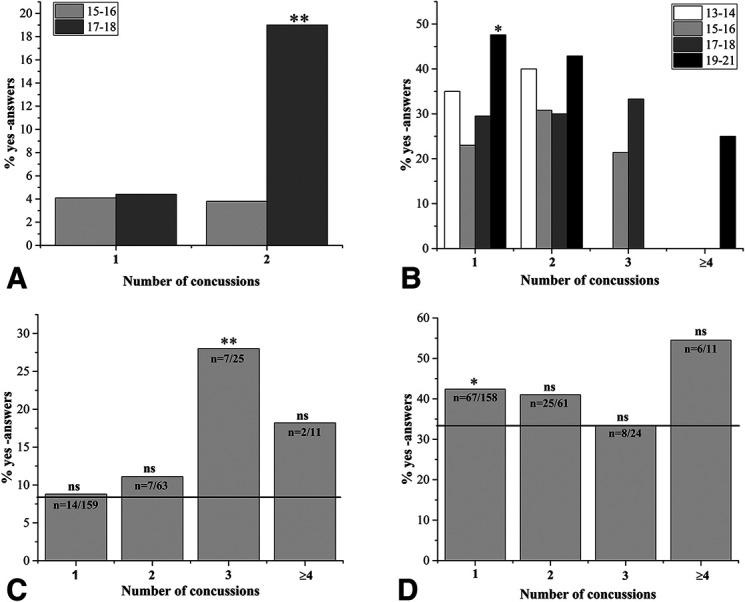
Statistically significant differences were observed in the answers to the functional vision questionnaire in 4 questions targeting general visual symptoms. Athletes with 1, 2, 3, or ≥4 concussions in their history were compared with age-matched athletes with no previous concussion history. Questions “*have you had problems seeing traffic signs or watching tv*” (**A**); “*do your eyes get tired when you read or use a computer*” (**B**); “*have you experienced frontal headaches*” (**C**); and “*do your eyes feel tired or strained at times*” (**D**) were answered “*yes”* or “*no”* by all athletes. The results from specific age groups (13–14 years, 15–16 years, 17–18 years, and 19–21 years) are shown in (**A** and **B**), and answers from all athletes are shown in (**C** and **D**). The reference line shown on (**C** and **D**) indicates the percentage of “*yes*” answers from all athletes with no concussion history. The *n* shown on the bars in (**C** and **D**) indicates the number of “*yes”* answers per total number of concussed athletes for each question. The *P*-values were calculated by comparing data with age-matched, nonconcussed athlete groups. ns, statistically nonsignificant, *P < 0.05, **P < 0.01.

Statistically significant increases in general vision symptoms were observed in the concussion history groups, as compared with the nonconcussed controls (Fig. 1). Significant problems with seeing traffic signs or watching TV were reported by the 17–18-year-old athletes with a history of 2 concussions (Fig. 1A). Eye fatigue was reported by all age groups, with significant increase found for 19–21-year-old athletes with 1 concussion (Fig. 1B). Among all players, the athletes with a history of 3 concussions reported significantly more frontal headaches (Fig. 1C), and eye fatigue was increased in athletes with 1 prior concussion (Fig. 1D).

**FIG. 2.**
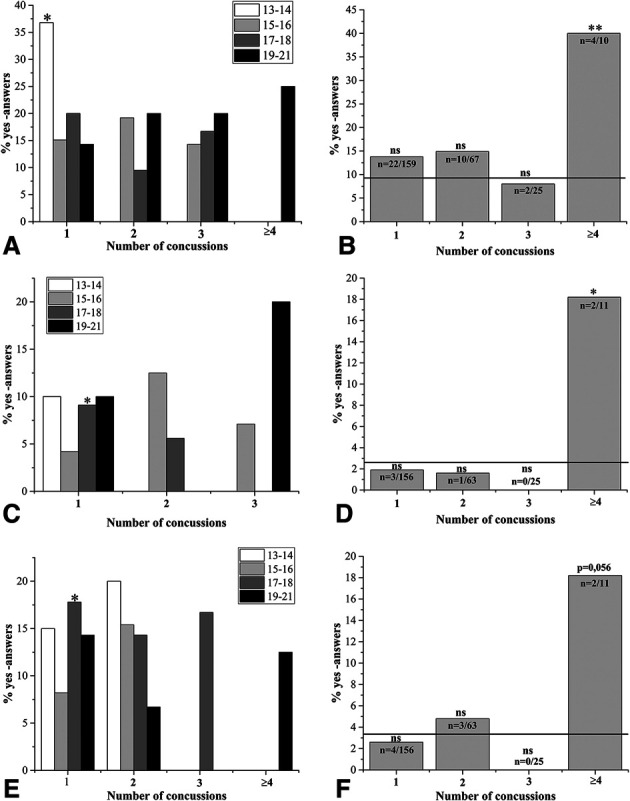
Accommodation and vergence disturbances were evaluated in the functional vision questionnaire by several questions. The results from specific age groups (13–14 years, 15–16 years, 17–18 years, and 19–21 years) are shown in (**A**, **B**, and **E**), and answers from all athletes are shown in (**C**, **D**, and **F**). Statistically significant increases in “*yes*” answers were observed for questions “*do you have to squint to see far better*” (**A**); “*do you at times lose the object in sight*” (**B**); and “*when watching far and near in turns, does it take a moment before the object is clear*” (**E**). When the data obtained from all athletes were evaluated, statistically significant increases in the percentages of “*yes*” answers were observed for the questions “*do you have to blink to make an unclear object clear*” (**C**) and “*have you ever had difficulties with depth perception or in evaluating distances*” (**D**). The increase in “*yes*” answers for the question “h*ave you ever noticed blurring or disappearing of the borders of field of sight during sport performance*” remained a bit under the significance threshold for the athletes with ≥4 concussions (**F**). The *n* shown on the bars in (**C**, **D**, and **F**) indicates the number of “*yes”* answers per total number of concussed players for each question. The reference line shown indicates the percentage of “*yes*” answers from all athletes with no concussion history. The *P*-values were calculated by comparing data with the age-matched, nonconcussed athlete groups. ns, statistically nonsignificant, *P < 0.05, **P < 0.01.

When eye-related compensatory symptoms were evaluated, statistically significantly increases in several symptoms were observed in the concussed athlete groups (Fig. 2). The increases in need for squinting (Fig. 2A), in feel of losing the object in sight (Fig. 2B), and blinking (Fig. 2C) suggest near triad disturbances. Together with the observed difficulties in depth perception (Fig. 2D), accommodation from far-to-near and vice versa (Fig. 2E), and blurring or disappearing of borders during sports (Fig. 2F) suggest that accommodation–vergence problems were relatively common in healthy athletes with concussion history.

**FIG. 3.**
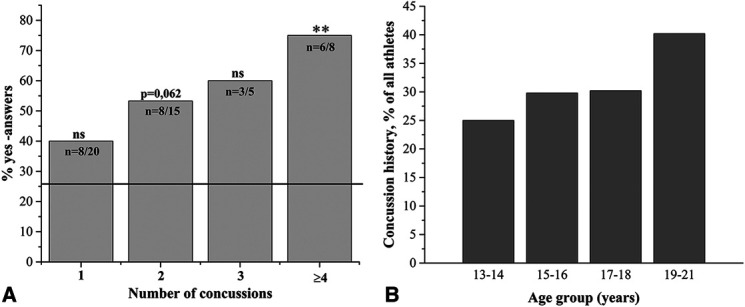
Integration of vision with cognitive operations was evaluated in the functional vision questionnaire by asking “*do you have problems concentrating when reading*” (**A**). An age-dependent increase in the concussion rate is shown in (**B**). The *n* shown on the bars in (**A**) indicates the number of “*yes”* answers per total number of 19–21-year-old concussed players. The reference line shown in (**A**) indicates the percentage of “*yes*” answers from age-matched athletes with no concussion history. The *P*-values were calculated by comparing data with an age-matched, nonconcussed athlete group. ns, statistically nonsignificant, **P < 0.01.

Reading is often used as a functional assessment of the integration of oculomotor functions, and reading problems may indicate vision problems. The 19–21-year-old athletes with ≥4 concussions had significantly more concentration problems when reading, suggesting difficulties in their cognitive functions (Fig. 3A). Also, concussion history increased with age (Fig. 3B).

## DISCUSSION

By definition, patients with concussion do not present significant morphologic brain pathology. Despite this, almost one-third of patients with acute concussion and more than three-fifth of patients with postconcussion syndrome have vestibulo-ocular dysfunctions. Owing to the multifactorial outcome of concussion-related symptoms, there are no validated evaluation or treatment protocols for prolonged vision symptoms specifically for athletes. The ocularmotor subtype of concussion causes difficulty with visual activities, asthenopia, problems with visual focus, blurred vision, frontal headaches, and difficulty judging distances—all symptoms that may lead to concentration problems. The interconnected “what,” “where,” and “how” visual pathways result in controlled and programmed movement actions. To facilitate this, rapid and accurate eye movements are imperative. The multiple nuclei and neural connections located in the midbrain and brainstem regulate horizontal and vertical gaze, lens accommodation, vergence, and pupil responses to light. Although problems with vision are common after concussions, the effects of concussion history on functional vision have remained unknown. In sports, vision problems may become a risk factor for concussion, because the ability to process rapidly alternating visual input is not optimal.

For an athlete, the demands on vision are high. For example, in ice hockey, the athlete must be able to continuously scan the field to keep the puck and other players in sight, while skating in high speed. It is, therefore, understandable that poor vision makes playing not only difficult but also dangerous. Visual skills significantly affect the on-ice game performance, and vision training might reduce head impact exposure in ice hockey. In this study, we addressed the ability to integrate and process visual information by a functional vision questionnaire that was specifically designed to find sport vision-related impairments. The major difference in our questionnaire related to other, clinically validated tools such as the NEI-VFQ-25^18^ is that we wanted to address vision in healthy, elite-level athletes. Therefore, we did not expect to find severe red flag signs suggesting midbrain pathology: neurobehavioral symptoms, cranial nerve palsies, visual field loss, or double vision. Instead, our questions specifically targeted visual perception during movement. When combined with neurocognitive performance, functional cognitive impairments were found to correlate with accumulating concussions.

Eye fatigue, concentration problems, and headaches observed here can become apparent for various reasons. Such symptoms are common acutely after concussion and within post-concussion syndrome patients,^7^ but the exact neuropathologic reasons behind the symptoms remain unknown. Convergence insufficiency and accommodation discrepancy may also cause frontal headaches.^19^ Our data indicated that frontal headaches were increased in athletes with a history of 3 concussions, which supports the idea that cumulative concussions may be a risk factor for more frequent headaches.

According to our findings, it seems that concussions and especially cumulative concussions correlate with impaired eye-specific compensatory mechanisms. It is generally accepted that normal visual function is fundamental for reading, and the problems in the visual system may lead to concentration problems. Athletes with a history of at least 4 concussions reported deficits in depth perception and evaluating distances. Furthermore, 17–18-year-old athletes with 1 concussion reported more of unclear images when watching far and near in turns, which may indicate a problem with accommodation, vergence, or a combination of both. This transient blurring may become evident because of accommodation insufficiency and slowness in changing the level of accommodation, which are sometimes described in patients with mild traumatic brain injury.^11^ Also, convergence insufficiency may cause this kind of problems. In addition, athletes with 2 concussions had more difficulties in seeing traffic signs or watching TV. Such actions are not merely enabled by accommodation and vergence, but it is clear that deficits therein have negative impacts on functional vision in sport. Blinking and squinting, eye-specific compensatory symptoms observed in athletes with concussion history, also indicated deficits in the visual system.^20^ Disappearing or blurring of field of sight, and losing the object in sight were probably caused by dysfunction of the vestibulo-ocular reflex, smooth pursuit, near triad, or a combination of all of these.

The participants of this study were elite-level athletes with high demands on their functional vision. On the basis of these results, accumulating concussions correlate with oculomotor impairments, and this may increase the injury risk of the athlete because of nonoptimal vision-dependent control of movement. Many of the vision problems reported by the healthy athletes could individually be considered minor. When combined with the requirements for visuospatial attention, timing, and movement control of elite sports, a questionnaire-based evaluation of functional vision may present as a cost-effective tool for injury prevention purposes.

STATEMENT OF AUTHORSHIP

Conception and design: L. Hokkanen, K. Peltonen, T. Laitala; Acquisition of data: K. Peltonen, T. Laitala; Analysis and interpretation of data: J. Leinonen, R. Mikkola, K. Peltonen, T. Laitala. Drafting the manuscript: J. Leinonen, T. Laitala; Revising the manuscript for intellectual content: L. Hokkanen, K. Peltonen, T. Laitala. Final approval of the completed manuscript: J. Leinonen, R. Mikkola, K. Peltonen, L. Hokkanen, T. Laitala.

## References

[1] The International Classification of Diseases 11th Revision. Available at: https://icd.who.int/en. Accessed April 28, 2024.

[2] Lefevre-Dognin C Cogné M Perdrieau V Granger A Heslot C Azouvi P. Definition and epidemiology of mild traumatic brain injury. Neuro Chirurg. 2021;67:218–221. 10.1016/j.neuchi.2020.02.002 32387427

[3] Rosene JM Raksnis B Silva B . Comparison of concussion rates between NCAA division I and division III men's and women's ice hockey players. Am J Sports Med. 2017;45:2622–2629. 28622025 10.1177/0363546517710005

[4] Lumba-Brown A Teramoto M Bloom OJ . Concussion guidelines step 2: evidence for subtype classification. Neurosurgery. 2020;86:2–13. 31432081 10.1093/neuros/nyz332 PMC6911735

[5] Hirad AA Bazarian JJ Merchant-Borna K . A common neural signature of brain injury in concussion and subconcussion. Sci Adv. 2019;5:eaau3460. 31457074 10.1126/sciadv.aau3460 PMC6685720

[6] Murray NG Szekely B Islas A . Smooth pursuit and saccades after sport-related concussion. J Neurotrauma. 2020;37:340–346. 31524054 10.1089/neu.2019.6595 PMC7059002

[7] Wiecek EK Roberts TL Shah AS Raghuram A. Vergence, accommodation, and visual tracking in children and adolescents evaluated in a multidisciplinary concussion clinic. Vis Res. 2021;184:30–36. 33838503 10.1016/j.visres.2021.03.002 PMC8145776

[8] Strominger M. Neuroanatomy and imaging assessment in traumatic brain injury. J Binocul Vis Ocul Motil. 2020;70:119–121. 33275075 10.1080/2576117X.2020.1730135

[9] Gallivan JP Goodale MA. The dorsal “action” pathway. Handb Clin Neurol. 2018;151:449–466. 29519474 10.1016/B978-0-444-63622-5.00023-1

[10] Romeu-Mejia R Giza CC Goldman JT. Concussion pathophysiology and injury biomechanics. Curr Rev Musculoskelet Med. 2019;12:105–116. 30820754 10.1007/s12178-019-09536-8 PMC6542913

[11] Visser K de Koning ME Ciubotariu D . An exploratory study on the association between blood-based biomarkers and subacute neurometabolic changes following mild traumatic brain injury. J Neurol. 2024;271:1985–1998. 38157029 10.1007/s00415-023-12146-7

[12] Master CL Bacal D Grady MF . AAP Section on Ophthalmology; American Academy of Ophthalmology; American Association for Pediatric Ophthalmology and Strabismus; and American Association of Certified Orthoptists. Vision and concussion: symptoms, signs, evaluation, and treatment. Pediatrics. 2022;150:e2021056047. 35843991 10.1542/peds.2021-056047

[13] Putukian M Purcell L Schneider KJ . Clinical recovery from concussion-return to school and sport: a systematic review and meta-analysis. Br J Sports Med. 2023;57:798–809. 37316183 10.1136/bjsports-2022-106682

[14] Zemek R Barrowman N Freedman SB , Pediatric Emergency Research Canada PERC Concussion Team. Clinical risk score for persistent postconcussion symptoms among children with acute concussion in the ED. JAMA. 2016;315:1014–1025. 26954410 10.1001/jama.2016.1203

[15] Master CL Master SR Wiebe DJ, et al. Vision and vestibular system dysfunction predicts prolonged concussion recovery in children. Clin J Sport Med. 2018;28:139–145. 29064869 10.1097/JSM.0000000000000507

[16] Patricios JS Schneider KJ Dvorak J . Consensus statement on concussion in sport: the 6th International Conference on Concussion in Sport-Amsterdam, October 2022. Br J Sports Med. 2023;57:695–711. 37316210 10.1136/bjsports-2023-106898

[17] Peltonen K Vartiainen M Laitala-Leinonen T . Adolescent athletes with learning disability display atypical maturational trajectories on concussion baseline testing: implications based on a Finnish sample. Child Neuropsychol. 2019;25:336–351. 29781392 10.1080/09297049.2018.1474865

[18] Khadka J McAlinden C Pesudovs K. Validation of the national eye institute visual function questionnaire-25 (NEI VFQ-25) in age-related macular degeneration. Invest Ophthalmol Vis Sci. 2012;53:1276. 22408138 10.1167/iovs.12-9541

[19] Nguyen E Inger H Jordan C Rogers D. Ocular causes for headache. Semin Pediatr Neurol. 2021;40:100925. 34749915 10.1016/j.spen.2021.100925

[20] Yang B Intoy J Rucci M. Eye blinks as a visual processing stage. Proc Natl Acad Sci U S A. 2024;121:e2310291121. 38564641 10.1073/pnas.2310291121 PMC11009678

